# Development of a Physiotherapist-Coordinated Interdisciplinary Rehabilitation Intervention for People with Suspected Axial Spondyloarthritis: The SPINCODE Rehabilitation Intervention

**DOI:** 10.3390/jcm13226830

**Published:** 2024-11-13

**Authors:** Kirsten Lykke Knak, Jette Primdahl, Georg Kröber, Camilla Fongen, John Graversgaard, Ann Bremander

**Affiliations:** 1 The Danish Centre for Expertise in Rheumatology, Danish Hospital for Rheumatic Diseases, University Hospital of Southern Denmark, 6400 Sønderborg, Denmark; 2 Department of Regional Health Research, University of Southern Denmark, 5000 Odense, Denmark; 3 Hospital Sønderjylland, University Hospital of Southern Denmark, 6200 Aabenraa, Denmark; 4 Center for Treatment of Rheumatic and Musculoskeletal Disease (REMEDY), Diakonhjemmet Hospital, 0319 Oslo, Norway; 5 Department of Clinical Sciences, Rheumatology Section, Lund University, 22148 Lund, Sweden; 6 Spenshult Research and Development Centre, 30274 Halmstad, Sweden

**Keywords:** complex intervention, multi-disciplinary, outpatient rehabilitation, low back pain, self-management, patient-centered care, Focused Acceptance and Commitment Therapy, coherence

## Abstract

**Background**: People with early axial spondyloarthritis experience a diagnostic delay and a similar disease burden as people with axial spondyloarthritis at a later stage of the disease. In many European countries, patients with early axial spondyloarthritis do not have access to an interdisciplinary rehabilitation team. The objective of this study was to develop a new evidence-based physiotherapist-coordinated interdisciplinary rehabilitation intervention for individuals suspected of axial spondyloarthritis. This development of the rehabilitation intervention is part of the SPINCODE project which focusses on early diagnosis and treatment for people with axial spondyloarthritis. **Methods**: The development of the intervention encompasses: (i) identifying the evidence base and program theories; (ii) modeling and remodeling the intervention; and (iii) describing the developed intervention. **Results**: The six-month SPINCODE rehabilitation intervention is a physiotherapist-coordinated, interdisciplinary, outpatient rehabilitation intervention at a specialized rheumatology hospital. The intervention consists of: (i) individual physiotherapist-coordinated consultations with assessment, goal setting, tailored physical activity support, and the defined goals, and coordination across the interdisciplinary team at the hospital and across primary and secondary healthcare levels; (ii) group sessions, encompassing patient education and peer support; and (iii) optional individual support from the interdisciplinary team. Physiotherapists from private care working with the patient enrolled in the SPINCODE study are offered digital support from the hospital-based physiotherapists. **Conclusions**: The developed physiotherapist-led interdisciplinary SPINCODE rehabilitation intervention is ready for feasibility testing.

## 1. Introduction

Low back pain (LBP) is a common condition, with a mean prevalence rate of 10,800 per 100,000 persons in Denmark [1]. Up to 1.5% of the general population have LBP because of axial spondyloarthritis (axSpA) [2], although the prevalence of axSpA is probably underestimated in people with LBP [3]. AxSpA is a chronic autoimmune disease, predominantly characterized by axial manifestations [4] and often presents in the third decade of life [5]. In the beginning, the disease presents with non-radiographic changes of the spine and the sacroiliac joints (nr-axSpA) [6]. In some patients, the disease develops into more advanced structural damage of the sacroiliac joints and spine (radiographic (r-)ax-SpA) [6]. The cardinal symptoms of axSpA are chronic pain and stiffness in the lower back and sacroiliac joints [6]. Other common disease features are arthritis, enthesitis, dactylitis [7], anterior uveitis, psoriasis or inflammatory bowel disease [8]. AxSpA is associated with an increased risk of comorbidities, such as osteoporosis, depression and cardiovascular diseases [9,10,11]. AxSpA can be debilitating for patients because of pain, fatigue [12], sleep problems [13], functional disability [14], impaired working capacity [15], and restrictions in their social life [16]. A similar disease burden has been demonstrated in people with either nr-axSpA and r-axSpA regarding pain, fatigue, functional disability, and reduced quality of life [17]. AxSpA has socioeconomic consequences for both the individual and society because of expensive drugs and reduced work capacity [18].

Diagnosing axSpA is challenging for health professionals [19], often leading to a diagnostic delay of up to 6.5 years in men and 8.8 years in women [20]. In an attempt to counteract this diagnostic delay, recommendations for early referral of patients with suspected axSpA have been made by the Assessment of SpondyloArthritis International Society (ASAS) [21]. The diagnostic process is long, with only one-third of true positive patients being reliably diagnosed within two years [22]. Patients with undiagnosed or non-radiographic axial spondyloarthritis (nr-axSpA) may experience a disease burden comparable to that of patients with radiographic axial spondyloarthritis (r-axSpA) [17]. Therefore, these individuals require both pharmacological and non-pharmacological support until a definitive diagnosis of axial spondyloarthritis (axSpA) is established or ruled out.

Despite advancements in pharmacological treatments, individuals with axSpA continue to experience a significant disease burden, even when low disease activity has been attained [23]. Thus, there is an ongoing need for rehabilitation for this patient population. The target of rehabilitation interventions is to optimize function and reduce disability, as opposed to focusing on the diagnosis itself [24]. The ASAS and the European Alliance of Associations for Rheumatology (EULAR) recommend a multidisciplinary approach to the management of people with axSpA, in which physiotherapy is an integral part of the treatment [25].

Exercise is a key component in the management of axSpA [25]. A recent meta-analysis [26] concluded that exercise has an effect on disease control and symptom relief in people with axSpA. In Denmark, at present, despite a comparable disease burden between individuals with nr-axSpA and r-axSpA, only those with r-axSpA have access to free physiotherapy, a policy that diverges from European recommendations [17]. Studies of interdisciplinary person-centered team rehabilitation, including physical exercise, education, and consultations with an interdisciplinary team [27,28,29,30], have shown positive effect on mobility [27,28], disease activity [27,28,29], physical function [28,29], pain [29], aerobic capacity [30], and well-being [29,30]. Outpatient rehabilitation of patients with axSpA can be suitable, given that the disease usually presents at the age when people are busy with their careers and family life. In addition, outpatient rehabilitation is less costly than inpatient rehabilitation [31,32]. The World Health Organization stresses the importance of coherent rehabilitation across sectors in the healthcare system [33]. To facilitate coherence across healthcare professionals and sectors, a coordinator is recommended [34,35]. Patient-centeredness [34], shared decision-making [25], and self-management [36] should be integral elements in rehabilitation offered to patients with inflammatory arthritis (IA), to empower patients in their everyday lives [36]. A single-arm study of an education and self-management program for patients with r-axSpA showed promising findings regarding decreased symptoms and disease activity, and increased quality of life [37]. To improve exercise behavior in people with axSpA, a behavioral approach was found useful [38].

We were not able to identify a long-term, physiotherapist-coordinated, interdisciplinary, outpatient, rehabilitation program to support self-management in people with suspected axSpA. Thus, we aimed to develop such an intervention in a context-specific setting.

The Spondyloarthritis Inception Cohort Study of Southern Denmark (SPINCODE) project is a 5-year prospective cohort study of individuals with low back pain of ≥3 months and at least one other feature supporting suspicion of axSpA (clinicaltrials.gov Identifier: NCT06337513). The SPINCODE project intends to decrease the diagnostic delay in individuals with axSpA and offer timely pharmacological and non-pharmacological treatment to these patients. The objectives of the SPINCODE project are (i) to investigate axSpA during the early course of disease and to compare the two groups (axSpA confirmed and axSpA not confirmed) at the end of the study (an observational cohort), and (ii) to develop, feasibility test, and investigate the effect of a new physiotherapist-coordinated interdisciplinary rehabilitation intervention (a clinical trial). One of the elements in the SPINCODE project is to raise awareness of axSpA in the primary care sector (physiotherapists, PTs, in private care and general practitioners) and among physicians treating axSpA-associated conditions, e.g., gastroenterologists, dermatologists, and ophthalmologists, by establishing educational meetings and online material for healthcare professionals. The educational initiatives also serve to recruit participants to the study and aid in increasing and easing referrals and reducing diagnostic delays of patients with symptoms suspicious of incipient axSpA.

Herein, this study’s objective was to develop a new evidence-based, physiotherapist-coordinated interdisciplinary outpatient rehabilitation intervention for individuals suspected of having axSpA, conducted as part of the SPINCODE project.

## 2. Materials and Methods

### 2.1. Study Design

**Figure 1:**
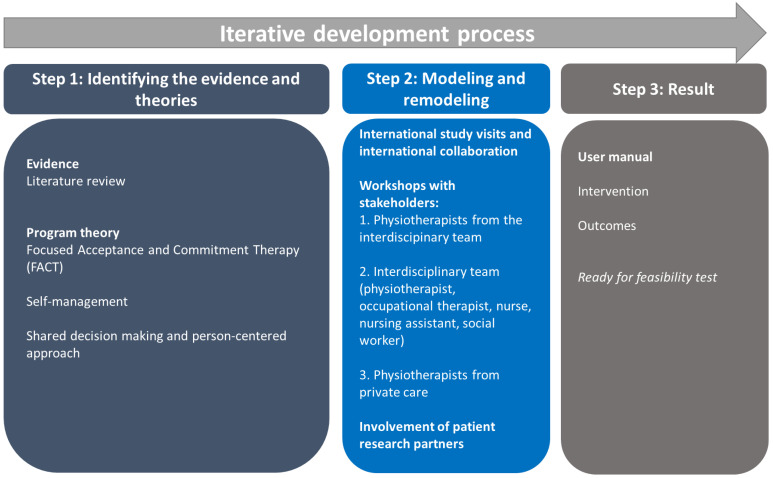
The development process of the SPINCODE rehabilitation intervention.

The SPINCODE rehabilitation intervention is part of the SPINCODE project, a cohort study with a focus on early diagnosis and treatment for people with early axSpA. The development of a novel rehabilitation intervention for people with suspected axSpA was planned in accordance with the UK Medical Research Council (MRC) framework for developing and evaluating complex interventions [39]. The overall recommended process from initiation to finalization of complex interventions is: 1. Development, 2. Feasibility testing, 3. Evaluation, and 4. Implementation of the intervention [39]. This paper addresses the first step “Development of an intervention”, and has taken the following core elements into consideration: context, program theory development and refinement, stakeholders, key uncertainties, intervention refinement, and economics [39]. The development process is described in the following stages: (i) identifying the evidence base and program theories, (ii) modeling and remodeling the intervention and outcomes, and (iii) reporting the result. The description of the intervention aligns with the Template for Intervention Description and Replication (TIDieR) guideline for reporting of trials [40] (please see Table S1). The SPINCODE rehabilitation intervention was developed in the period from April 2022 to December 2023 (Figure 1).

### 2.2. Intervention Development

#### 2.2.1. Identifying the Evidence Base and Selecting the Program Theories

A literature search was made for published systematic reviews and EULAR recommendations over the last ten years, regarding non-pharmacological interventions for patients with spondyloarthritis (SpA). There were 48 hits on the PubMed database from the following search string on systematic reviews: ((“inflammatory arthritis”[Title] OR spondyl*[Title]) AND (systematic review [Publication Type])) AND (“non-pharmacological treatment” OR exercise OR rehabilitation OR “physical activity”) with a 10-year filter. There were 106 hits on the PubMed database from the following search string on EULAR recommendations: (EULAR recommendations spondyloarthritis) with a 10-year filter. The focus of the data extraction was recommendations for non-pharmacological interventions and theories to support behavior change in patients with SpA (see Table 1).

Three program theories were identified to underpin the intervention: (i) a cognitive behavioral therapy approach: Focused Acceptance and Commitment Therapy (FACT), (ii) self-management, and (iii) shared decision making/a person-centered approach (Table 2).

#### 2.2.2. Modeling and Remodeling

**Figure 2:**
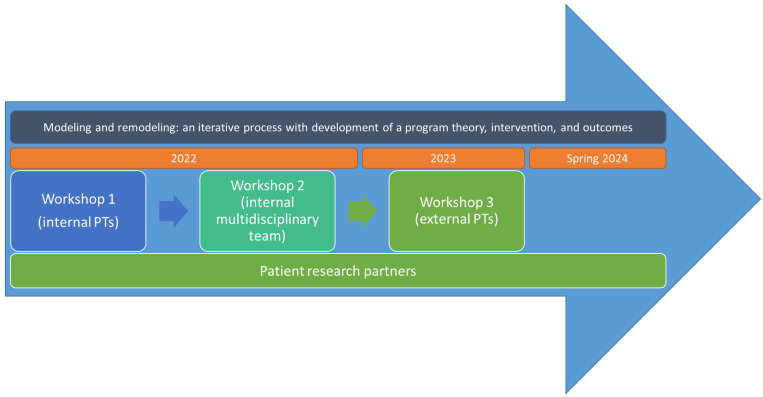
Involvement of key stakeholders in the development of the SPINCODE rehabilitation intervention. PT = physiotherapist.

Key stakeholders were involved in the development process, including healthcare professionals at the Danish Hospital for Rheumatic Diseases (DHRD) (manager of the physiotherapy and occupational therapy department, PTs, occupational therapists, OT, nurses, a nursing assistant, and a social worker), PTs from private care in the surrounding municipalities, researchers from the research group, and three patient research partners (PRP, including the co-author J.G.) (Figure 2).

#### Workshops

Three workshops involving key stakeholders were planned and conducted from spring 2022 to spring 2023. All workshops were facilitated by two moderators, the first author (KK) and the last author (AB). Workshop 1 (PTs at DHRD) and workshop 2 (interdisciplinary team at DHRD) were held face-to-face at DHRD. At both workshops, the participants were encouraged to write notes on sticky notes with their reflections and ideas. The moderators took notes, and the workshops were audio-recorded after written informed consent was obtained. Workshop 3 (PTs from private care) was held online, out of respect for the private practice PTs’ working schedule.

Workshop 1 was a 2 h workshop held in May 2022 at DHRD. Seven PTs and the manager of the physiotherapy department participated. The workshop encompassed a brief presentation of the preliminary SPINCODE design, followed by discussions in smaller groups, and finally a plenary discussion. As physical activity is a core treatment in patients with axSpA, the intervention will be led by PTs and, thus, the aim was to discuss the content of the intervention with the PTs. Moreover, the aim was to discuss collaboration with PTs from private care in the four geographically close municipalities in the Region of Southern Denmark. The private PTs might be interested in professional sparring with rheumatology PTs regarding patients suspected with axSpA who are involved in the intervention during the intervention period. Highlights of the workshop are provided in Table 3.

Workshop 2 was planned as a 2 h workshop in October 2022 at DHRD. The participants consisted of two PTs, one social worker, one OT, one nursing assistant, and two nurses, all experienced in rehabilitation interventions. The structure of the workshop was a presentation of the preliminary design, followed by discussions in pairs and a plenary discussion at the end. The aim of this workshop was to be inspired by experiences from previous, similar interdisciplinary research projects at DHRD [69,70] regarding overall management, interdisciplinary team work, patient education, and outpatient interventions to inform and optimize the content of the complex PT-coordinated interdisciplinary rehabilitation intervention. Highlights of the workshop are provided in Table 3.

Workshop 3 was a 1.5 h online session in April 2023. Five PTs from private care in one of the four municipalities of Southern Denmark participated. The agenda was a short presentation of the project, followed by a discussion of the following pre-defined themes: 1. Thoughts on referral of patients with possible axSpA, and 2. Thoughts on an opportunity to have a hotline to PTs at DHRD. Highlights of the workshop are provided in Table 3.

#### Feedback from the Patient Research Partners (PRPs)

We aimed to recruit at least one male patient and one female patient with possible early axSpA < 45 years as PRPs. Three PRPs agreed to be part of the SPINCODE research team (males, age 36 to 59, all diagnosed with psoriatic arthritis or SpA with a disease duration of 3 to 11 years). All PRPs were invited to an introduction meeting with researchers from the SPINCODE project. The PRPs were introduced to the aim of the SPINCODE project, and mutual expectations and clarification of the individual needs of each PRP regarding practicalities (response time, physical or online meetings, etc.) were discussed.

Highlights from the workshop are provided in Table 3. In addition to feedback on the draft rehabilitation intervention and possible outcomes, all the PRPs provided feedback on the participant information letter, online patient education materials provided by the interdisciplinary team, and the development of a pamphlet for the participants in SPINCODE rehabilitation intervention, which included information on physical activity in patients with chronic low back pain (knowledge of chronic low back pain and physical activity, recommendations on physical activity, behavior change to increase physical activity, physical exercises, and own notes).

#### International Study Visits and International Collaboration

The SPINCODE rehabilitation intervention design was discussed with healthcare professionals from abroad, including Professor Emma Dures from School of Health and Social Wellbeing at University of the West of England, Bristol, UK, co-author and PT Camilla Fongen (CF) from the Center for Treatment of Rheumatic and Musculoskeletal Disease (REMEDY), Diakonhjemmet Hospital, Oslo, Norway. The first author visited Diakonhjemmet Hospital in the Spring 2022 and again in 2023, where the project was discussed with CF and other researchers from the REMEDY, who are specialized in non-pharmacological treatment of patients with RMDs, including axSpA.

#### Outcome Selection

**Figure 3:**
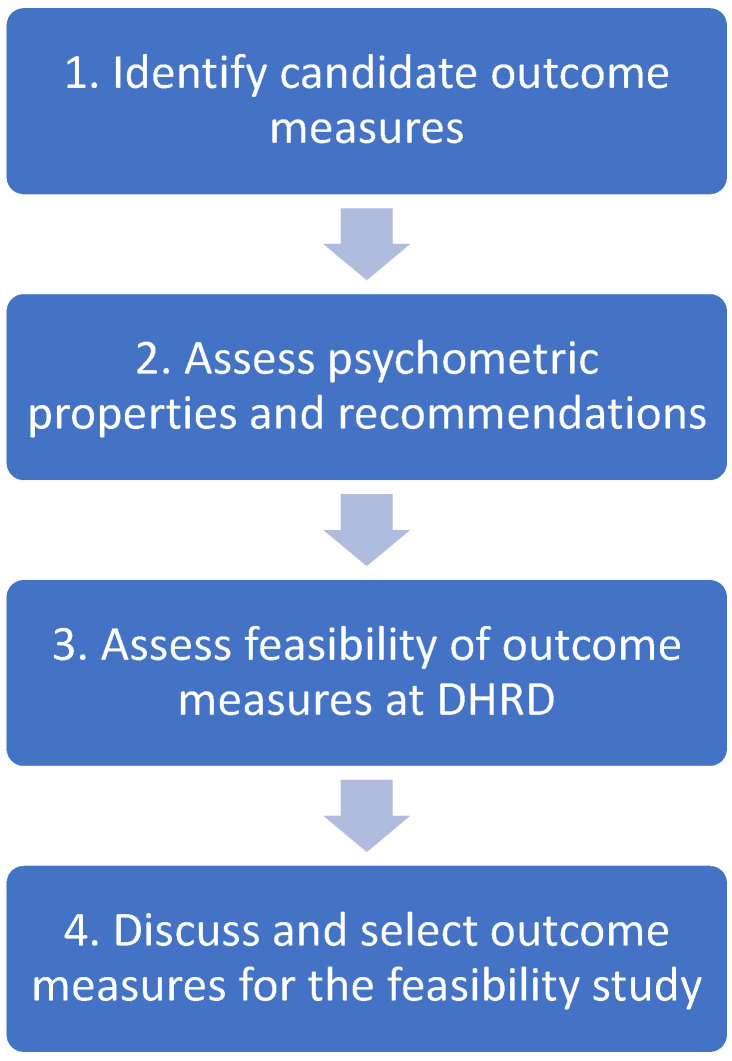
The selection process for outcome measures in the SPINCODE rehabilitation intervention. DHRD = Danish Hospital for Rheumatic Diseases.

The selection of outcome measures in the present study was made by the research group, informed by input from the stakeholders as described in Table 3 and depicted in Figure 3. Evaluation of outcome measures (including psychometric properties) is described in Table 4. Adverse events will be registered in the Danish rheumatology quality database (DANBIO) during the consultations with the coordinating PT in the rehabilitation intervention or during consultation with the rheumatologist as part of the SPINCODE project. Additionally, as part of the SPINCODE project, the Bath Ankylosing Spondylitis Metrology Index (BASMI), including thorax expansion, will be assessed by the rheumatologist, data available for the SPINCODE rehabilitation team through DANBIO.

Given that the SPINCODE project spans five years and each participant will be monitored for two years, with follow-up by a physiotherapist every six months following the initial six-month rehabilitation intervention, there is a substantial risk that the same assessor/examiner/or team member may not be available for all assessments of a given participant. Therefore, we conducted a study of the inter-rater reliability of the ASPI test between two raters in patients with axSpA at DHRD (in preparation) [96]. To minimize outcome measurement error and enhance the coherence of the rehabilitation intervention, efforts were made to ensure that each participant was monitored by a single coordinating PT. Accordingly, the number of PTs involved and the PTs’ duty schedules were adjusted.

A rehabilitation manual describing the detailed content and outcome measures was modeled and continuously remodeled, based on feedback from the PRPs and the interdisciplinary team.

## 3. Results

### 3.1. Content of the SPINCODE Rehabilitation Intervention

**Figure 4:**
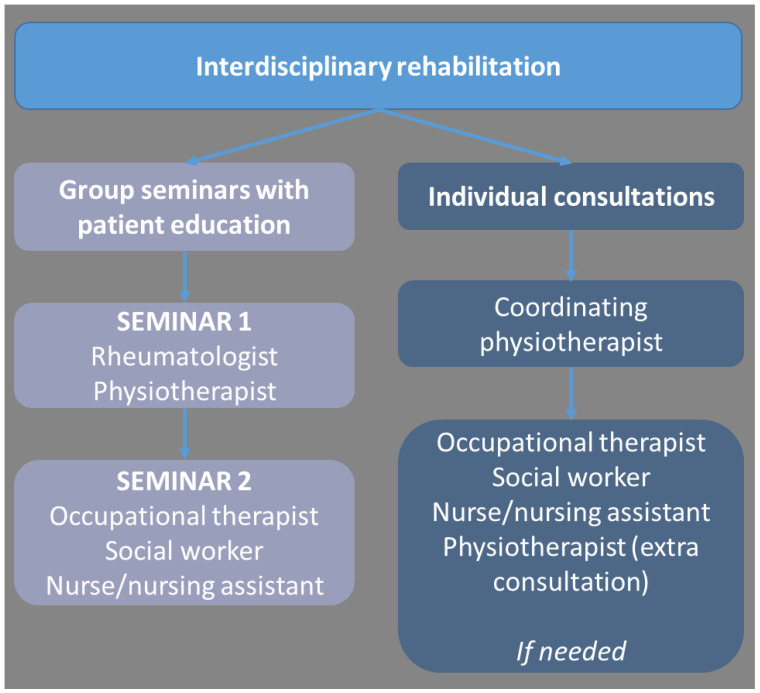
Content in the SPINCODE interdisciplinary rehabilitation intervention.

The final six-month SPINCODE rehabilitation intervention encompasses two mandatory patient education group seminars, with 8–10 participants in each group, three individual consultations with the coordinating PT, and elective needs-based individual consultations with various HCPs from the interdisciplinary team, if needed (a maximum of six hours) (Figure 4). As part of the SPINCODE project, the patient is monitored by a rheumatologist during rehabilitation and receives medical treatment according to national guidelines [97].

**Figure 5:**
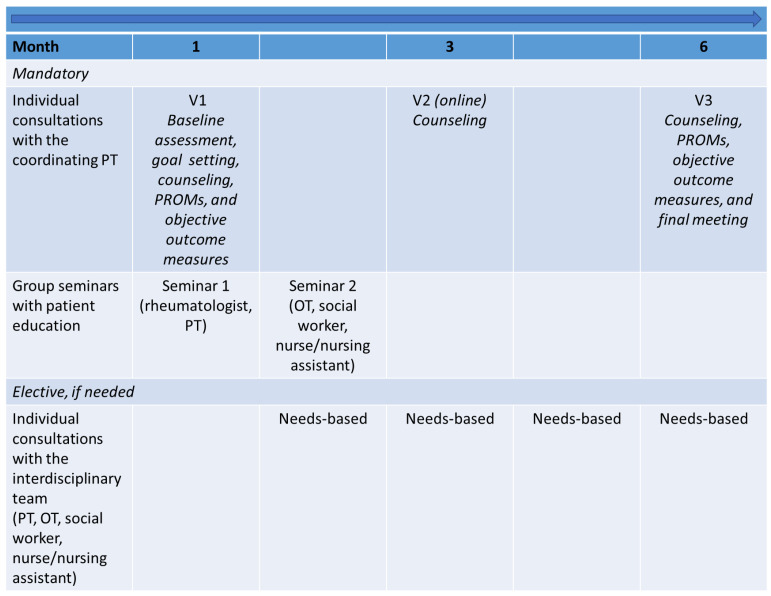
Timeline of the SPINCODE rehabilitation intervention. OT = occupational therapist, PROM = Patient-Reported Outcome Measure, PT = physiotherapist, V1 = visit 1, V2 = visit 2, V3 = visit 3.

A timeline of individual consultations and group seminars in the SPINCODE intervention is visualized in Figure 5, and a detailed description of the rehabilitation content is provided in Table 5.

**Figure 6:**
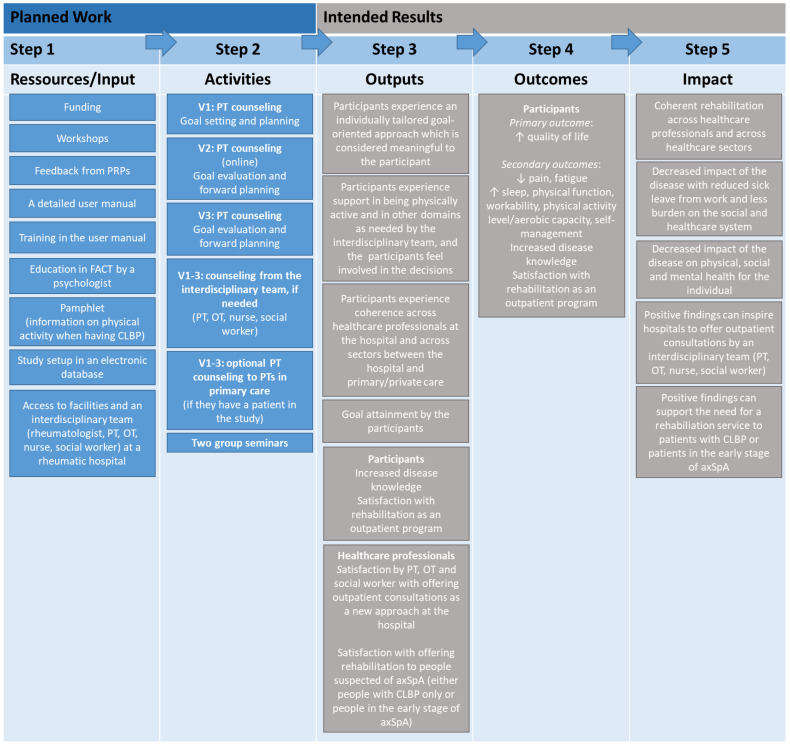
The logic model for SPINCODE rehabilitation intervention. axSpA = axial spondyloarthritis, CLBP = chronic low back pain, FACT = Focused Acceptance and Commitment Therapy, OT = occupational therapist, PRP = patient research partner, PT = physiotherapist, V1 = visit 1, V2 = visit 2, V3 = visit 3.

In the manual, supportive worksheets are provided regarding bio-psycho-social assessments and questions, relevant initiatives in the four municipalities of Southern Denmark, and description of opportunities for support by each HCP in the interdisciplinary team. Additionally, the manual includes supportive worksheets for the physical assessments (pelvic provocation tests [101], ASPI [62], and indirect maximal aerobic capacity testing using the modified Balke protocol on a treadmill [64]). The group seminars have 8–10 participants and the seminars are scheduled late afternoon/early evening, given that the participants are expected to be in paid work. A logic model was developed to describe the relation between the planned work (resources and activities) and the intended results from the intervention (outputs, outcomes, and long-term impact) [102]. A logic model is provided for the SPINCODE rehabilitation intervention in Figure 6.

### 3.2. Objective and Patient-Reported Outcome Measures

The selected outcomes will be tested in a feasibility study and evaluated before possible inclusion in a later planned RCT. The outcome measures chosen are shown in Table 4.

### 3.3. A Subsequent Feasibility Study

The developed SPINCODE rehabilitation intervention is currently being tested in a rheumatology outpatient clinic at the DHRD, in accordance with the MRC framework [39].

#### 3.3.1. Target Population

The target population is adults (≥18 years of age) with suspected axSpA with symptom onset at age ≤ 45, back pain ≥ 3 months, diagnosed with anterior uveitis (AU) and/or psoriasis and/or inflammatory bowel disease (IBD) and/or HLA-B 27 positive, and/or imaging suspicious for axSpA.

#### 3.3.2. Competence Development

To ensure competences of the clinical staff who are to deliver the intervention, competence development was planned. The HCPs were invited to a 3 h meeting with a presentation of the SPINCODE rehabilitation intervention manual in December 2023, where the central theoretical concepts were presented and discussed. The HCPs were asked to read the manual beforehand. Furthermore, ongoing ad hoc meetings were held with the interdisciplinary team regarding discussion of the online patient educational material, guideline for referral to HCPs from the interdisciplinary team and introduction to use DANBIO (a national rheumatology quality database) [103]. Three training sessions of 1.5 h duration have been conducted for the PTs to learn how to use the objective physical outcome measures in the study. The HCPs at DHRD are trained in FACT during four sessions of 3 h duration by a psychologist followed by the opportunity for monthly 2 h group-based supervision from a social worker or psychologist. In addition, the HCPs have experience with interdisciplinary teamwork, goal setting based on shared decision making and previous participation in development and research projects at the DHRD.

In the feasibility study, we will evaluate recruitment, retention, fidelity, data collection, number of telephone contacts from PTs in private care and number of individual consultations with each member in the interdisciplinary team. In addition, we will explore possible tendency for changes in the outcome measures. Moreover, interviews with the participants and HCPs participating in the SPINCODE intervention will be planned and conducted to explore their experiences. If the results from the feasibility study are promising, the SPINCODE rehabilitation intervention will be adapted based on the results and the adapted version will be tested in an RCT. The SPINCODE rehabilitation intervention is approved by the Regional Committees on Health Research Ethics (project-ID S-20230055).

## 4. Discussion

The objective of this study was to develop an interdisciplinary PT-coordinated rehabilitation intervention in an outpatient setting for patients referred to the DHRD with suspicion of an axSpA disease, in alignment with the MRC framework [39]. Relevant stakeholders and evidence informed the development. The overall content of the intervention consists of individual consultations with the coordinating PT, two patient seminars in groups, and individual needs-based consultations with the interdisciplinary team. The overarching focus of the intervention is to support physical activity, self-management, and facilitate coherence across HCPs at the DHRD and across healthcare sectors to achieve increased quality of life.

The SPINCODE rehabilitation was developed according to the MRC framework establishing evidence and theories, involving key stakeholders, modeling and re-modeling the intervention, similar to previous studies developing complex outpatient rehabilitation interventions to patients with inflammatory arthritis [69,70,104]. In alignment with previous studies [69,70,104], the present study included self-management [69,70,104], self-efficacy [69,70,104], shared decision making [70], and a cognitive behavioral therapy approach [69,104] as program theories. There may be other relevant program theories which could underpin the SPINCODE intervention, including health literacy which considers the patient’s ability to find, read, understand, reflect and act on health information [69,105], and occupational balance to balance daily activities, including paid work, housekeeping, and leisure activities [106]. However, the selected theories (FACT, shared decision making, patient centeredness, self-management, and self-efficacy) were deemed highly relevant by both the clinicians and the researchers and were prioritized as key theories in the present study.

The HCPs raised concerns about the risk of medicalization of the participants with suspected axSpA for whom the axSpA diagnosis will be disconfirmed during the trial period. Therefore, this is a special attention point in the SPINCODE rehabilitation intervention. Referral as needed to the interdisciplinary team is in alignment with the ASAS/EULAR recommendation of interdisciplinary management in patients with axSpA [25].

According to the MRC framework [39], key uncertainties and economics should be considered. Because there is no economic coverage for outpatient consultations with a PT, OT or social worker in rheumatology clinics in most Danish regions, political decisions are needed to make the intervention feasible and economically viable. In many European countries, people with suspected axSpA do not have access to a rehabilitation team, which makes it more difficult to implement positive results internationally. Key uncertainties will be evaluated in the feasibility study and encompass:

- Recruitment: recruitment regarding sufficient referral of patients with suspected axSpA.
- Administration and content: Coordination of the rheumatologist and PT appointments in the SPINCODE project, availability of booking appointments at the PT department, access to the same PT at repeated testing, available time appointments in case of participant or HCP illness, whether the scheduled timeframe for the visits is sufficient and perceived as meaningful by the participants and HCPs, whether the HCPs feel competent to deliver the intervention content and within the scheduled timeframe, and effectiveness of the intervention on outcome measures.
- Coordination: Coordination across HCPs at DHRD and across healthcare sectors, consultation, and communication with PTs in private care.
- Patient seminars: Preparation of online material before patient seminars, and whether the seminars are perceived as meaningful by the participants and PTs.

### Strengths and Limitations

It is considered a strength that the intervention was developed in accordance with the MRC framework and underpinned by program theories. In addition, the intervention is based on current evidence, and key stakeholders have been involved in the development process, including PRPs, HCPs from the interdisciplinary team (PT, OT, nurse/nursing assistant, and social worker), and PTs from the private care of Southern Denmark. Moreover, the development process and feasibility test are anchored at the DHRD, where possible positive results can be implemented. Hence, translation from research to clinic is facilitated. The involved HCPs are all rheumatology specialists, and several have experience in rehabilitation and other previous and ongoing research projects at the DHRD.

Outcome measures in the intervention included PROMs, which are subjective and may be influenced by recall and social desirability bias (e.g., physical activity level). Some PROMs can only be assessed subjectively, e.g., HRQoL. Other concepts, e.g., physical mobility and physical activity, can also be assessed by physical performance-based tests. Hence, the PROMs in the rehabilitation intervention were supplemented with physical performance-based tests to measure spinal mobility (BASMI), physical function (ASPI), and aerobic capacity (the modified Balke protocol) (Table 4). Since the rehabilitation visits (V1 + V3) are time-consuming because of PROMs and physical performance tests in addition to counselling, the PROMs and physical performance tests were limited and key measures were prioritized. Additionally, the nature of certain symptoms, such as fatigue, pain, and sleep disturbances, may fluctuate over time. Despite our initiatives to recruit a group of PRPs that would include both male and female patients and preferably with early axSpA < 45 years, all PRPs were male, with an age span of 36 to 59. We seek to include an additional one to two female patients with early axSpA for the analysis and interpretation of results from the initiated feasibility study.

## 5. Conclusions

The objective of this study was to develop a PT coordinated interdisciplinary outpatient SPINCODE rehabilitation intervention for people with LBP suspected of axSpA as part of the SPINCODE project with early diagnosis and treatment for people with axSpA. The intervention was developed based on the MRC framework for complex interventions, establishing the evidence and identifying the program theories, modeling and remodeling, and presenting the results. The development resulted in a six-month PT-coordinated interdisciplinary rehabilitation intervention with three individual consultations with the coordinating PT, two patient education seminars in groups with the interdisciplinary team, and a maximum of six individual consultations with the interdisciplinary team if needed. In addition, the participants are followed by a rheumatologist regarding axSpA diagnostic and pharmacological treatment. The SPINCODE rehabilitation intervention is currently being tested in a feasibility study. If the results are promising, the rehabilitation intervention will be adapted as needed and the efficacy will be tested in an RCT.

## Figures and Tables

**Table 1:** Overview of EULAR recommendations and systematic review findings in people with axSpA.

Themes	Recommendations
Non-pharmacological interventions for SpA	A combination of pharmacological and non-pharmacological management is recommended [25,41]. Regular physical activity and exercise (strength and aerobic exercise) of moderate [42] or moderate to vigorous intensity [43] are recommended. Various types of exercises can have an effect on disease activity [26,42,43,44,45,46,47], physical function [42,43,44,46,47], pain [26,42,43,44,45], mobility [44,46,47], cardiorespiratory function [44], depression [44], and quality of life [42,44]. Aerobic exercise is recommended to decrease cardiovascular risk [48] and can have a positive impact on disease activity, physical function, and cardiorespiratory function [49,50], albeit possible adverse events should be considered [51]. Physical activity can be beneficial for quality of life [51], physical function [51], cardiorespiratory function [52], muscle strength [52], while physical inactivity should be avoided [42]. Supervised group exercise is recommended over unsupervised home exercise [53]. Patient education is recommended [25,41,45,54].
Theories to support behavior change in IA	Self-management [36,54], patient-centered approach [36,54], and shared decision making [54] are recommended.

IA = inflammatory arthritis, nr-axSpA = non-radiographic axial spondyloarthritis, r-axSpA = radiographic axial spondyloarthritis, SpA = spondyloarthritis.

**Table 2:** Program theories supporting the SPINCODE rehabilitation intervention.

Theoretical Approaches	Overall Description
Focused Acceptance and Commitment Therapy (FACT)	FACT is a short version of cognitive behavioral therapy tailored to clinical practice with limited available time. The overall aim of FACT is psychological flexibility and to support a person to change or adapt a behavior to what is beneficial in the long term and in alignment with one’s life values. FACT consists of four elements: (i) *Values*: awareness of a person’s life values and status on whether the person is living according to one’s life values. Life values should be the determinant factor for one’s behavior. (ii) *Functionality*: a neutral non-judging exploration of a person’s present behavior, its long-term consequences and how to initiate a behavior consciously aligned with one’s life values. (iii) *Defusion and acceptance*: acceptance of a person’s life situation that is unchangeable (e.g., a chronic disease) is fundamental to avoid spending energy on things you want to escape from. Instead, one should spend energy on the things one wants. Defusion promotes a flexible mindset, where the person recognizes that one’s thoughts are not the truth and should not determine one’s behavior—a person’s behavior should be navigated by one’s values. (iv) *Self-compassion*: self-compassion is treating oneself with solicitude and acknowledging that behavioral change is difficult. Instead of punishing oneself, a person should act on difficulties and lack of successes in a self-compassionate and constructive way [55].
Self-management and self-efficacy	Self-management is a person’s ability to manage his/her symptoms, treatment, and biopsychosocial consequences of living with a chronic disease [56]. Patients should be empowered to manage life with a disease and be active actors in their own life, e.g., making well-informed decisions, applying relevant resources, and asking for help when needed [57]. EULAR stresses the importance of incorporating self-management in the management of people with inflammatory arthritis [36]. Self-management is closely linked to self-efficacy. Self-efficacy is a person’s confidence in their own abilities to solve specific problems or challenges and is modulated by four sources: (i) performance accomplishment (own successes), (ii) vicarious experiences (others’ successes), (iii) verbal persuasion (others’ confidence in you), and (iv) emotional arousal (e.g., palpitations) [58].
Shared decision making and a person-centered approach	Shared decision making is collaboration and communication between the healthcare provider and the patient to reach a decision [59]. The process of shared decision making consists of: (i) team talk: patient preferences and information provided by the healthcare professional, (ii) option talk: discussion of alternatives, pros and cons, and (iii) decision talk: informed patient preferences and decisions [59]. Shared decision making is one of the overarching principles in the ASAS-EULAR recommendations for the management of axSpA [25].A person-centered approach involves active engagement in decision making [60] and is closely related to shared decision making. A person-centered approach means that the intervention is focused on the patient and tailored to the patient’s values [61].

ASAS = Assessment of SpondyloArthritis international Society, EULAR = European Alliance of Associations for Rheumatology, FACT = Focused Acceptance and Commitment Therapy.

**Table 3:** Highlights from stakeholders in the development process of the SPINCODE rehabilitation intervention.

Stakeholders	Highlights
PTs from the DHRD (workshop 1)	
Theme: content of a PT-coordinated rehabilitation program	The participants believed that each participant’s life values are essential for rehabilitation, and that counseling should be individually tailored. An ethical dilemma was highlighted, as the participants wanted to avoid medicalization of the participants with suspected axSpA, given that only a minority of them would be diagnosed with the disease at a later stage. The participants addressed attention to safety, if PTs were to use maximal aerobic capacity test and the need for training of PTs in the clinic to be able to perform the tests that were selected in accordance with guidelines.
Theme: collaboration with PTs from private care	A range of opportunities for collaboration across secondary and primary care were discussed: PTs at DHRD training PTs from private care, a dialogue meeting, an online information platform, and PTs at DHRD having a consultant function for PTs in private care.
Interdisciplinary staff from the DHRD (workshop 2)	
Theme: experience from previous inter-disciplinary research projects at DHRD—what worked well and less well? Informing the development of SPINCODE	The participants highlighted the value of interdisciplinary group sessions for patient education with peer support and close teamwork in the interdisciplinary team. The participants stressed that patient education sessions should be interactive. In the case of referral to other clinicians in the team, a specific goal for the referral was required. The importance of a coordinator was emphasized.
PTs from private care (workshop 3)	
Theme: referral of patients suspected of axSpA	Various opportunities to raise awareness of people with possible axSpA was discussed (meetings, networks, physiotherapy professional journal). An online available checklist for anamnesis and assessment was requested, to evaluate an axSpA suspicion. It was mentioned that PTs are unable to directly refer patients to rheumatologists, which was seen as a barrier.
Theme: hotline to PTs at DHRD	The participants supported the suggestion of a telephone hotline to the PTs at DHRD.
Patient research partners	
Theme: patient-reported outcome measures (PROMs) and design of the rehabilitation intervention	The number of PROMs was problematized. Sufficient time for the PT consultation was emphasized, to ensure valuable counseling A coordinating PT, peer support, and additional support if needed were recommended.
Input from international advisors and literature review	
Theme: outcome measures	Recommendation to use the objective Ankylosing Spondylitis Performance Index (ASPI) [62] as a substitute for or supplement to the less responsive, subjective Bath Ankylosing Spondylitis Functional Index (BASFI) questionnaire [63]. Practical demonstration of ASPI and indirect maximal aerobic capacity testing on a treadmill, using the modified Balke protocol [64]. The relevance of aerobic capacity testing in people with axSpA, after screening for contraindication [65], was stressed because of the disease-associated increased risk of cardiovascular complications [10]. Aerobic capacity testing using the Balke protocol is safe in patients with AS [66], and maximal aerobic capacity testing has a higher precision than has submaximal aerobic capacity testing [67].
Theme: referral of patients with possible axSpA	The need for an ongoing awareness campaign targeted to the referring healthcare professionals in primary and secondary care was emphasized, based on experiences from the SPondyloArthritis Caught Early (SPACE) cohort [68].

ASPI = Ankylosing Spondylitis Performance Index, axSpA = axial spondyloarthritis, AS = ankylosing spondylitis, BASFI = Bath Ankylosing Spondylitis Functional Index, DHRD = Danish Hospital for Rheumatic Diseases, PROM = patient-reported outcome measure, PT = physiotherapist, SPACE = SPondyloArthritis Caught Early.

**Table 4:** Evaluation of the selected outcome measures in the SPINCODE rehabilitation intervention.

	Domain	Specific Test	Reason for Selection
Primary outcome	Health related quality of life	EuroQol 5 dimension 5 level version (EQ-5D-5L) [71].	Overall functioning and health are mandatory domains of the ASAS-OMERACT core set for patients with axSpA [72]. EQ5D is correlated with Ankylosing Spondylitis Quality of Life questionnaire (ASQoL) (*r* = 0.71) [73] and is available in Danish, in contrast to ASQoL.
Secondary outcomes	Pain	Pain NRS from Bath Ankylosing Spondylitis Disease Activity Index (BASDAI) [74].	Pain is a mandatory domain in the ASAS-OMERACT core set for patients with axSpA [72].
	Spinal mobility	Bath Ankylosing Spondylitis Metrology Index (BASMI) [75].	Spinal mobility is an important but optional domain in the ASAS-OMERACT core set for patients with axSpA [72].
	Morning stiffness	Bath Ankylosing Spondylitis Disease Activity Index (BASDAI) [74].	Morning stiffness is a mandatory domain in the ASAS-OMERACT core set for patients with axSpA [72].
	Disease activity	Bath Ankylosing Spondylitis Disease Activity Index (BASDAI) [74].	Disease activity is a mandatory domain in the ASAS-OMERACT core set for patients with axSpA [72].
	Fatigue	Bristol Rheumatoid Arthritis Fatigue Numeric Rating Scales for severity, impact and coping version 2 (BRAF-NRS v2) [76].	Fatigue is a mandatory domain in the ASAS-OMERACT core set for patients with axSpA [72]. BRAF-NRS v2 is validated in patients with axSpA (paper in preparation) [77].
	Sleep	Insomnia Severity Index (ISI) [78].	Sleep is an important but optional domain in the ASAS-OMERACT core set for patients with axSpA [72]. ISI has been shown to be valid, reliable, and sensitive in patients with insomnia [78], is shorter than the Pittsburgh Sleep Quality index (PSQI) [79], and is available in Danish.
	Patient acceptability	Patient Acceptable Symptom State (PASS) [80].	PASS has been validated in patients with AS [81].
	Anxiety/depression	Items from (EQ-5D-5L) [71].	Overall functioning and health are mandatory domains in the ASAS-OMERACT core set in patients with axSpA [72]. Anxiety and depression are prevalent in patients with axSpA [9].
	Workability	Work Productivity and Activity Impairment Questionnaire (WPAI) (four metrics: Absenteeism, Presenteeism, Overall work productivity loss, and Activity impairment) [82].	Work is an important but optional domain in the ASAS-OMERACT core set for patients with axSpA [72]. WPAI is valid [83,84], reliable [83,84], and responsive [84] in patients with axSpA.
	Self-management	Self-Efficacy for Managing Chronic Diseases 6-item Scale (SES6G) [85].	SES6G focuses on living with a chronic disease and has been found to be valid and reliable in patients with chronic diseases [85].
	Physical function	Bath Ankylosing Spondylitis Functional Index (BASFI) (PROM) [86]. Ankylosing Spondylitis Performance Index (ASPI) (performance based test) [62].	Physical functioning is a mandatory domain in the ASAS-OMERACT core set for patients with axSpA [72]. The objective ASPI is reliable [62,87,88] and more responsive than the subjective BASFI in patients with SpA [63].
	Physical activity level	Physical Activity Scale version 2 (PAS2) [89].	The psychometric evidence for subjective questionnaires on physical activity is relatively weak [90,91,92,93]. The modified SQUASCH is valid, reliable, and responsive in patients with axSpA [94] but is not available in Danish.
	Aerobic capacity	Indirect maximal aerobic capacity test by the modified Balke protocol on a treadmill [66,95] (a priori screening for contraindications and approval by a rheumatologist).	Direct maximal capacity testing is superior to indirect maximal testing, but direct maximal capacity testing is more time-consuming and the equipment is unavailable at DHRD and most rheumatology clinics.
Safety outcome	Adverse event	Registration of adverse events. In the SPINCODE rehabilitation intervention, events such as muscle pain, injuries and falls during physical activities will be tracked at planned or needs-based PT consultations. There is a team in place to adjudicate those incidents (a rheumatologist, the coordinating PT, and a research nurse).	Adverse event is a mandatory domain in the ASAS-OMERACT core set for patients with axSpA [72].

AS = Ankylosing Spondylitis, ASAS-OMERACT = Assessment of SpondyloArthritis international Society-Outcomes Measures in Rheumatology, ASPI = Ankylosing Spondylitis Performance Index, ASQoL = Ankylosing Spondylitis Quality of Life questionnaire, BASDAI = Bath Ankylosing Spondylitis Disease Activity Index, BASFI = Bath Ankylosing Spondylitis Functional Index, BASMI = Bath Ankylosing Spondylitis Metrology Index, BRAF-NRS v2 = Bristol Rheumatoid Arthritis Fatigue Numeric Rating Scales version 2, EQ-5D-5L = EuroQol-5-dimension 5 level-version, ISI = Insomnia Severity Index, PASS = Patient Acceptable Symptom State, PAS2 = Physical Activity Scale version 2, PROM = Patient-Reported Outcome Measure, PSQI = Pittsburgh Sleep Quality index, SES6G = Self-Efficacy for Managing Chronic Diseases 6-item Scale, WPAI = Work Productivity and Activity Impairment Questionnaire.

**Table 5:** The content of the SPINCODE rehabilitation intervention.

	Aim	Content
Coordinating PT		
Visit 1—initial consultation (1.75 h)	To introduce the intervention and align expectations. To perform a bio-psycho-social assessment, objective physical assessments, goal setting, and individually tailored counseling. Ensure coherence across HCPs at DHRD and across healthcare sectors.	The coordinating PT informs about the rehabilitation intervention and aligns intervention expectations. The PT performs an initial bio-psycho-social assessment including objective physical tests. Up to five activities that are perceived as troublesome by the participant are defined using PSFS [98], and goals are set based on shared decision making. Person-centered counseling is provided based on the agreed goal, the defined activities in the PSFS, the program theories (FACT, shared decision making, person-centeredness, self-management), and physical activity recommendations (see Table 1). If the participant is consulting a PT in private care besides participating in the SPINCODE project, this PT can contact the coordinating PT if the participant agrees. The PT supports the participant in identifying and engaging in relevant provision in the municipality or local community. A SPINCODE physiotherapy pamphlet with information on physical activity for low back pain (recommended behavior, physical activity, specific exercises, behavior change, links to further information) is offered.
Visit 2—continuous consultation (0.5 h) (online)	To continue the individually tailored counseling, and to ensure goal achievement and coherence across HCPs at DHRD and across healthcare sectors.	The PT evaluates the bio-psycho-social status and progress on the defined activities from the PSFS and goal attainment. Goal setting for the next visit is planned together with the participant. Person-centered counseling based on the bio-psycho-social assessment, agreed activities and goals, program theories (FACT, shared decision making, person-centeredness, self-management), and physical activity recommendations [25,99,100]. The PT supports the participant in identifying and engaging in relevant services in the municipality or local community. In collaboration with the participant, the PT coordinates referrals to interdisciplinary team members (PT, OT, nurse/nursing assistant, social worker) if needed.
Visit 3—final consultation (1.25 h)	To continue the individually tailored counseling, to ensure goal achievement and coherence across HCPs at DHRD and across healthcare sectors, and to plan the next steps after the intervention.	The PT evaluates the participant’s bio-psycho-social status, PROMs, objective physical outcomes, and progress on the defined activities from PSFS and goal attainment. Person-centered counseling based on the findings, agreed goals, program theories (FACT, shared decision making, person-centeredness, self-management), and physical activity recommendations [25,99,100]. The PT supports the participant in finding and initiating relevant services in the municipality or local community. Goals and the next steps after this 6-month intervention are planned.
Group seminars		
Seminar 1 (2.5 h)	To enhance self-management by providing patient education on axSpA, pharmacological and non-pharmacological interventions for axSpA delivered by a PT and rheumatologist and to facilitate peer support	Seminar 1 is conducted by a PT and a rheumatologist. The participants are requested to watch online materials developed by the healthcare professionals before the seminar. The content of the online material is: PT The benefit of physical activity/exercise in patients with axSpA or chronic LBP; Exercise, pain, inflammation, and cardiovascular risk. Rheumatologist Knowledge about axSpA; Pharmacological treatment of axSpA.The seminar is interactive and participant-driven, including questions and themes the participants want elaboration on based on the content of the online material.
Seminar 2 (2.5 h)	To enhance self-management by providing patient education on disease management in everyday life delivered by an OT, nurse/nursing assistant, and social worker and to facilitate peer support	Seminar 2 is conducted by an OT, nurse/nursing assistant, and a social worker. The participants are requested to watch online materials developed by the healthcare professionals before the seminar. The content of the online material is: OT Disease management with a focus on work, daily activities, and energy management.Nurse/nursing assistant Management of pain, fatigue, sleep, and understanding of and living with a chronic disease. Social worker Legislation regarding potential support within the social, educational, and labor market areas for those with a chronic disease. The seminar is interactive and participant-driven, including questions and themes the participants want elaboration on based on the content of the online material.
Individual consultations with the interdisciplinary team (if needed)		
PT (0.5 h)	To provide individual self-management support on physical activity and exercise.	Support additional to the existing PT consultations for goal achievement is provided, to address new problems, or additional support for, e.g., a shoulder issue or Achilles tendinitis in connection with axSpA. For physical problems not related to LBP or axSpA or not related to goal achievement, the participant is recommended to contact her/his general practitioner.
OT (0.5 h)	To provide individual self-management support on work and daily activities.	Individual support regarding energy management, management of daily activities, positioning, sleep, and assistive devices.
Nurse/nursing assistant (0.5 h)	To provide individual self-management support on pain, fatigue, sleep, and understanding of chronic disease.	Individual support regarding living with a chronic disease, the pharmacological treatment, and management of fatigue, pain, and sleep problems.
Social worker (1.0 h)	To provide individual self-management support or support within the social, educational, and labor market areas.	Individual support for supportive initiatives within exercise, education, and employment according to the legislation.

axSpA = axial spondyloarthritis, DHRD = Danish Hospital for Rheumatic Diseases, FACT = Focused Acceptance and Commitment Therapy, h = hour, HCP = healthcare professionals, LBP = Low Back Pain, OT = occupational therapist, PROM = Patient-Reported Outcome Measure, PSFS = Patient Specific Functional Scale, PT = physiotherapist, V1 = visit 1, V2 = visit 2, V3 = visit 3.

## Data Availability

Data are contained within the article.

## Supplementary Materials

The following supporting information can be downloaded at: https://www.mdpi.com/article/10.3390/jcm13226830/s1, Table S1: Template of the Intervention and Replication Checklist for SPINCODE.
