# Supplementary material for: Development of a Physiotherapist-Coordinated Interdisciplinary Rehabilitation Intervention for People with Suspected Axial Spondyloarthritis: The SPINCODE Rehabilitation Intervention
Source: J Clin Med. 2024 Nov 13;13(22):6830. doi: 10.3390/jcm13226830 (PMC11595147; doi:10.3390/jcm13226830)
Supplement: Supplementary file 1 [file jcm-13-06830-s001.zip › jcm-3228692-supplementary.pdf]

Supplementary File

Table 1. Template of the Intervention and Replication Checklist for SPINCODE

Item	Description
Intervention name	A complex physiotherapist-coordinated interdisciplinary rehabilitation intervention for people with suspected axial spondyloarthritis: the SPondyloarthritis INception COhort of southern DEnmark (SPINCODE) study
Rationale	Physiotherapist-coordinated interdisciplinary rehabilitation may have an effect on quality of life, physical function, aerobic capacity, physical activity level, sleep, fatigue, pain, workability, anxiety, depression, and self-efficacy in people with suspected axial spondyloarthritis.<br><br>The theoretical approaches underpinning the study consist of Focused Acceptance and Commitment Therapy (FACT), self-management, and person-centered approach/shared decision making.<br><br>*FACT* is a short version of cognitive behavioral therapy tailored to clinical practice with limited available time. The overall aim of FACT is psychological flexibility and to support a person in a behavioral change, with the intention of adapting a behavior that is beneficial in the long term and in alignment with the one's life values.<br>FACT consists of four elements:<br>(i) Values: awareness of a person's life values and status on whether the person is living according to one's life values. Life values should be the determining factor for one's behavior<br>(ii) Functionality: a neutral non-judging exploration of a person's present behavior, its long-term consequences and how to initiate a behavior that is consciously aligned with one's life values<br>(iii) Defusion and acceptance: acceptance of a person's life situation that is unchangeable (e.g., a disease) is fundamental to avoid spending energy on things one wants to escape from, and instead spend energy on things one wants. Defusion promotes a flexible mindset where the person recognizes that one's thoughts are not the truth and should not determine one's behavior – a person's behavior should be navigated by one's values.<br>(iv) Self-compassion: self-compassion is treating oneself with solicitude and acknowledging that behavioral change is difficult. Instead of punishing oneself, a person should act on difficulties and lack of successes in a self-compassionate and constructive way.<br>[55]

	Self-management is a person's ability to manage his/hers symptoms, treatment, and biopsychosocial consequences of living with a chronic disease [56]. Patients should be empowered to manage their life with a disease and be active actors in their own life, e.g., making well-informed decisions, applying relevant resources, and ask for help when needed [57]. The EULAR organization has stressed the importance of incorporating self-management in the management of people with inflammatory arthritis [36]. Self-management is closely linked to self-efficacy. Self-efficacy is a person's confidence in their own abilities and is modulated by four sources: (i) performance accomplishment (own successes), (ii) vicarious experiences (others' successes), (iii) verbal persuasion (others' confidence in you), and (iv) emotional arousal [58].<br><br>*Shared decision making and person-centered approach. Shared decision making* is collaboration and communication between the healthcare provider and the patient [59]. The process of shared decision making comprises: (i) team talk: patient preferences and information provided by the healthcare professional; (ii) option talk: discussion of alternatives, pros and cons; and (iii) Decision talk: informed patient preferences and decisions [59]. Shared decision making is one of the overarching principles in the ASAS-EULAR recommendations for the management of axSpA [25]. *A person-centered approach* involves active engagement in decision making [60] and is thus closely related to shared decision making. A person-centered approach means that the intervention is focused on the patient and tailored to the patient's values [61].<br><br>*Skills at DHRD:*<br>HPs take a person-centered approach and are experienced in shared decision making and self-management. The HPs are trained in FACT [55], which is a brief intervention for radical behaviour change.<br><br>Furthermore, the PTs at DHRD are trained in performing the ASPI test and aerobic capacity testing, using the modified Balke protocol.
Materials	A detailed manual supports HPs who deliver the intervention in SPINCODE. The manual encompasses: aim of the intervention, background, screening and inclusion/exclusion criteria, theories behind the intervention, content and practicalities of individual PT sessions, group seminars, and individual sessions with HPs from the multidisciplinary team, if needed, counseling to PTs from private care, outcome measures, templates, and worksheets for physical tests.<br><br>Participants are offered a SPINCODE physiotherapy pamphlet with information on physical activity when they have CLBP.
Training	The HPs received a detailed manual and were invited to a 3-hour meeting with an introduction to the manual. The HPs at DHRD had previously received training in FACT across four modules, each lasting

	three hours in duration. The PTs received training sessions of 1.5 hours duration regarding the objective physical outcome measures to be used in the study.
Coordinating PT	*Visit 1 – initial consultation (1.75 h):*<br>The coordinating PT informs the participant about the rehabilitation intervention and aligns intervention expectations. The PT performs an initial bio-psycho-social assessment, including objective physical tests. Up to five activities, that are perceived as troublesome by the participant, is defined using Patient Specific Functional Scale (PSFS) and goals are set based on shared decision making. Person-centered counseling is provided based on the agreed goal, the defined activities in the PSFS, the program theories (FACT, shared decision making, person-centeredness, self-management), and physical activity recommendations. If the participant is consulting a PT in private care, besides participating in SPINCODE, that PT can contact the coordinating PT, if the participant agrees. The PT supports the participant in finding and initiating relevant initiatives in the municipality or local community. In collaboration with the participant, the PT coordinates referral to the interdisciplinary team (PT, OT, nurse, social worker), if needed.<br>The PT hands out a SPINCODE physiotherapy pamphlet with information on physical activity for low back pain (recommended behavior, physical activity, specific exercises, behavior change, links to further information).<br><br>*Visit 2 – continuous consultation (0.5 h) (online):*<br>The PT evaluates the bio-psycho-social status and progress on the defined activities from the PSFS and goal attainment. Goal setting for the next visit is planned together with the participant. Person-centered counseling based on the findings, agreed goals, program theories (FACT, shared decision making, person-centeredness, self-management), and physical activity recommendations. The PT supports the participant in finding and initiating relevant services in the municipality or local community. A need for referral to the interdisciplinary team (PT, OT, nurse, social worker) is coordinated, if needed.<br><br>*Visit 3 – final consultation (1.25 h):*<br>The PT evaluates the participant's bio-psycho-social status, PROMs, objective physical outcomes, and progress on the defined activities from the PSFS and goal attainment. Person-centered counseling based on the findings, agreed goals, program theories (FACT, shared decision making, person-centeredness, self-management), and physical activity recommendations. The PT supports the participant in finding and initiating relevant services in the municipality or local community. Goals and the next steps after this 6-month intervention are planned.

	PTs from private care working with the client enrolled in the SPINCODE study are offered digital support from the PTs at DHRD.
Group seminars	The participants participate in two group seminars at the beginning of the study (before visit 2 with the coordinating PT). Before the seminars, the participants are asked to watch online education materials developed by the HPs. Each seminar has a duration of 2.5 hours, and the group size is 8-10 participants. The overall aim of the seminars is patient education and peer support.<br><br>*Seminar 1:*<br>Physiotherapist:<br>-the benefit of physical activity/exercise in patients with axSpA or chronic LBP<br>-Exercise, pain, inflammation, and cardiovascular risk<br><br>Rheumatologist:<br>-Knowledge about axSpA<br>-Pharmacological treatment of axSpA<br><br>*Seminar 2:*<br>Occupational therapist:<br>-Disease management with a focus on work, daily activities, and energy management<br><br>Nurse/nursing assistant:<br>-Management of pain, fatigue, sleep, and understanding of and living with a chronic disease<br><br>Social worker:<br>-Legislation regarding potential support within the social, educational, and labour market areas when having a chronic disease
Individual consultations (*additional*)	If needed, individual consultations with the relevant HPs from the multidisciplinary team (social worker, nurse/nursing assistant, OT, and additional sessions with a physiotherapist) are offered after the group seminars.<br><br>*PT:*<br>Additional support for the existing PT consultations for goal achievement is provided, to address new problems, or additional support regarding, e.g., a shoulder issue or Achilles tendinitis linked to axSpA. For physical problems not related to LBP or axSpA or not related to goal achievement, the participant is recommended to contact her/his general practitioner.<br><br>*OT:*

	Individual support regarding energy management, management of daily activities, positioning, sleep, and assistive devices.<br><br>*Nurse:*<br>Individual support regarding living with a chronic disease, the pharmacological treatment, and management of fatigue, pain, and sleep problems.<br><br>*Social worker:*<br>Individual support regarding legislation as to exercise subsidies, education, and labour market for those with a disease.
Delivery	SPINCODE is delivered at DHRD as an outpatient intervention. Visit 2 with the PT is by phone or online. Additional consultations with HPs from the multidisciplinary team can be in person, online (using Zoom) or by phone, depending on what the participant prefers.
Duration and dose	SPINCODE has a duration of 6 months and consists of 3 consultations with the coordinating PT, 2 group seminars, and the opportunity to have additional individual consultations with HPs from the multidisciplinary team, if needed (a maximum of 6 hours).

Abbreviations: Health professionals (HP), Danish Hospital for Rheumatic Diseases (DHRD), focused acceptance and commitment therapy (FACT), ankylosing spondylitis performance index (ASPI), SPondyloarthritis INception COhort of southern DEnmark (SPINCODE), axial spondyloarthritis (axSpA), chronic low back pain (CLBP), European Alliance of Associations for Rheumatology (EULAR), Assessment of SpondyloArthritis international Society (ASAS), Patient Specific Functional Scale (PSFS)
